# Vignette studies of medical choice and judgement to study caregivers' medical decision behaviour: systematic review

**DOI:** 10.1186/1471-2288-8-50

**Published:** 2008-07-30

**Authors:** Lucas M Bachmann, Andrea Mühleisen, Annekatrin Bock, Gerben ter Riet, Ulrike Held, Alfons GH Kessels

**Affiliations:** 1 Horten Centre, University of Zurich, Bolleystrasse 40, CH-8091 Zurich, Switzerland; 2 Division of Epidemiology and Biostatistics, Department of Social and Preventive Medicine, University of Bern, Switzerland; 3 Department of General Practice, Academic Medical Center, Amsterdam, The Netherlands; 4 Department of Clinical Epidemiology and Medical Technology Assessment, Maastricht University Hospital, Maastricht, The Netherlands

## Abstract

**Background:**

Vignette studies of medical choice and judgement have gained popularity in the medical literature. Originally developed in mathematical psychology they can be used to evaluate physicians' behaviour in the setting of diagnostic testing or treatment decisions. We provide an overview of the use, objectives and methodology of these studies in the medical field.

**Methods:**

Systematic review. We searched in electronic databases; reference lists of included studies. We included studies that examined medical decisions of physicians, nurses or medical students using cue weightings from answers to structured vignettes. Two reviewers scrutinized abstracts and examined full text copies of potentially eligible studies. The aim of the included studies, the type of clinical decision, the number of participants, some technical aspects, and the type of statistical analysis were extracted in duplicate and discrepancies were resolved by consensus.

**Results:**

30 reports published between 1983 and 2005 fulfilled the inclusion criteria. 22 studies (73%) reported on treatment decisions and 27 (90%) explored the variation of decisions among experts. Nine studies (30%) described differences in decisions between groups of caregivers and ten studies (33%) described the decision behaviour of only one group. Only six studies (20%) compared decision behaviour against an empirical reference of a correct decision. The median number of considered attributes was 6.5 (IQR 4–9), the median number of vignettes was 27 (IQR 16–40). In 17 studies, decision makers had to rate the relative importance of a given vignette; in six studies they had to assign a probability to each vignette. Only ten studies (33%) applied a statistical procedure to account for correlated data.

**Conclusion:**

Various studies of medical choice and judgement have been performed to depict weightings of the value of clinical information from answers to structured vignettes of care givers. We found that the design and analysis methods used in current applications vary considerably and could be improved in a large number of cases.

## Background

Preferences and perceived similarities or differences between choice alternatives can be evaluated using structured vignettes. There are two prominent methods of constructing such models of medical judgements, each with their own literature and set of advocates. These are conjoint analysis, developed in the 1970s to study preference and choice [1], and judgement analysis, also called social judgement theory, developed in the 1950s from Brunswik's lens model [2,3]. The two have developed along very different theoretical lines and have developed somewhat different methodology, although there is considerable overlap. Today there is a large number of marketing applications, where the joint effects of multiple product attributes on product choice have been studied. The types of choices include 'ranking', 'rating', and 'discrete choice'.

These methods can be carried forward to the analysis of medical decision making, as medical decisions require judgement under uncertainty. This uncertainty may concern a state, such as the presence of illness, the likelihood of future events, such as those in the natural course of an illness, or the likelihood with which such events may be averted, that is, treatment effects. For many years decision-making research has explored physicians' estimation of probabilities given clinical scenarios [4]. However, there have been concerns whether physicians' probability setting leads to consistent ratings [5]. Moreover, cognitive psychological research shows that physicians do not apply probabilities as suggested by decision-making theory but use their own heuristics to decide [6-8].

Studies of medical choice and judgement offer a way to elicit the public's, patients' and caregivers' views on healthcare that circumvents probability statements [9-11]. The technique is gaining widespread use in healthcare and has been applied in different areas for example to establish patients' preferences in the doctor-patient relationship [12], or to determine optimal treatments for patients [13]. Increasingly, discrete choice analyses are being employed to study how physicians weigh clinical information in the diagnostic work-up. In particular, respondents are asked to rank, rate, or choose between simulated clinical cases varying in values of different symptoms along the possibility that this case will have a certain illness or will need a certain treatment. Comparison with the results of clinical studies allows an analysis of potential discrepancies (e.g. undervaluation of signs and symptoms, overvaluation of test results). Moreover, such comparisons with reference data from clinical studies allow linking physicians' behaviour to illness probabilities and therefore allow examining (implicit) decision thresholds.

A considerable number of studies have been published recently. We provide an overview of existing reports, present an inventory of their objectives and methods, and evaluate them using systematic review methodology.

## Methods

We defined a study of medical choice and judgement as an investigation in which preferences were elicited in physicians, nurse practitioners or medical students and that allowed the estimation of the relative importance of different characteristics.

### Search strategy

We performed electronic searches in Medline, PsychINFO, CINAHL (Ovid^®^-version). Web of Science (ISI web of Science^®^) was used to locate studies that cited four key papers [14-17]. The last update search was performed on 25/3/2005. The exact search strategy may be obtained from the authors.

### Inclusion criteria

Eligible articles for this review had to infer cue or attribute weighting from answers to structured vignettes and had to report on caregivers' decision making.

### Data extraction strategy

We developed a data extraction form based on the assessment of three articles [17-19]. The form contained twelve items describing a study's salient features of context, design and analysis (for details see Table 1).

**Table 1:** Salient features of studies included in the systematic review.

**Author**	**Year of publication**	**Clinical problem**	**Type^1 ^of decision**	**Aim^2 ^+ reference^2^**	**Number of participants**	**Type^3 ^of participants**	**Number of vignettes**	**Number of attributes**	**Source^4 ^of attributes**	**Type^5 ^of outcome**	**Type^6 ^of analysis**
Kirwan	1983	Rheumatoid arthritis	3	3	2	4	17	5	2	2	2
Wigton	1986	Pulmonary embolism	1	5 → B	55	4, 1	27	8	1	6	1
Smith	1987	Tube feeding	2	2	222	4, 1	12	6	1	2	2
Holmes	1989	Hypertension	1	3	98	3	16	4	1	2	1
Von Preyss-Friedman	1992	Tube feeding	2	2	141	4, 4	16	6	1	2	1
Lee	1994	Surgical patients	2	2	34	4, 3, 2	30	8	4	2	1
Harries	1996	Diversity of diseases	2	3	32	4	130	13	3	2	1
McKinlay	1997	Breast cancer	4	1	128	4	32	6	1	2, 4	1
Shea	1997	Bile duct stones	4	2	624	4, 4	27	8	1	1, 2, 4	1
Skaner	1998	Heart failure	1	1	27	4	40	10	1	1	2
Timmermans	1997	Colonic emergency	2	2	102	4	16	3	1	2	1
VanMilten-burg-Van Zijl	1997	Unstable angina	2	2	18	4, 4	12	7	2	2	2
Ross	1999	Depression	4	1	407	4	6	2	3	2, 4	1
Backlund	2000	Hypercholesterolemia	2	5 → A	38	4	40	8	2	2	1
Haggerty	2000	Fetal risk situation	3	1	573	2	32	10	1	2	1
Skaner	2000	Heart failure	1	5 → C	70	4, 4, 1	40	8	2	1	2
Bouma	2001	Aortic stenosis	2	2	275	4, 4	32	10	9	2	1
Engelsbel	2001	Ectopic pregnancy	1	4	27	4	16	6	9	2	2
Kee	2002	Renal disease	2	1	8	4	50	11	2	2, 3	1
Sorum	2002	Acute otitis media	4	2	75	4, 4	46	15	3	1, 2	2
Sorum	2002	Acute otitis media	4	4	75	4, 4	46	15	1	1, 4	2
Wahlström	2002	Asthma	2	5 → A	314	4, 4, 4	18	5	3	2	1
Bouma	2004	Aortic stenosis	2	5 → B	34	4	32	9	9	2	1
Sorum	2003	Prostate cancer	4	2	65	4, 4, 4	32	5	1	1, 2	2
Tamayo-Sarver	2003	Opioid analgesic	2	1	2872	4	3	3	3	4	1
Mays	2004	Vaccine program	2	1	224	2	13	4	9	2	1
Raley	2004	Papilloma vaccine	2	1	181	4	13	4	9	2	1
Arnold	2005	Resp. tract infection	2	1	257	4, 4	16	4	1	2	1
Lee	2005	Postoperat. recovery	5	1	60	4, 3, 2	8	3	3	5	2
Tiemeier	2002	Depression	2	5 → A	449	4, 4, 4	22	7	3	4, 6	1

1) 1 = diagnosis, 2 = treatment, 3 = risk, prognosis, 4 = diagnosis & treatment, 5 = other

2) 1^st ^digit describes aim: 1 = descriptive, 2 = group comparison, 3 = consistency, 4 = change over time, 5 = comparison with reference

2^nd ^digit describes reference: A = guidelines, B = actual patients, C = clinical study

3) 1 = student, 2 = paramedic, 3 = physician in training, 4 = expert

4) 1 = literature, 2 = patients, 3 = expert, 4 = guidelines, 5 = no information

5) 1 = probability, 2 = rating, 3 = ranking, 4 = yes/no choice, 5 = discrete choice, 6 = >2 alternatives

6) 1 = no adjustment for correlated data, 2 = adjustment for correlated data

Besides some study descriptors such as first author and year of publication, we extracted information on the studies' objectives, the clinical problem, who the decision-maker was, the type of decision/preference (diagnosis, treatment, risk, prognosis, diagnosis & treatment, and other), the number of participants and the authors' aims. The objectives were extracted into five categories: description of preferences in one group of caregivers (1), comparison of two or more groups such as different professions or different levels of competence. (2), assessment of the consistency within caregivers with their actual decisions or their direct rating of the attributes (3), assessment of changes in preferences over time, e.g. after attending a course (4), and comparison of caregivers with guidelines (5a), actual patients' preferences (5b), or the findings of one or more clinical studies (5c). We also registered the number of vignettes, the number of attributes of each vignette and the rationale behind the selection of the attributes. Finally, we documented how participants were asked to respond to the vignettes: rating (yes/no, otherwise), ranking, probability estimates, or discrete choice and the way, if any, in which authors accounted for correlated data in the analysis. We extracted this item because observations resulting from these experiments are typically not independent. Each respondent evaluates each of the vignettes. This makes the data from one respondent more alike than one would expect under the assumption of independence, and therefore standard deviations of the attributes could be underestimated. We searched for any statistical method that allows to adjust the standard errors for the intra-group correlation.

All studies were assessed in duplicate. Discordant scores based on reading errors were corrected. Discordant scores based on real differences in interpretation were discussed and resolved through consensus.

## Results

**Figure 1:**
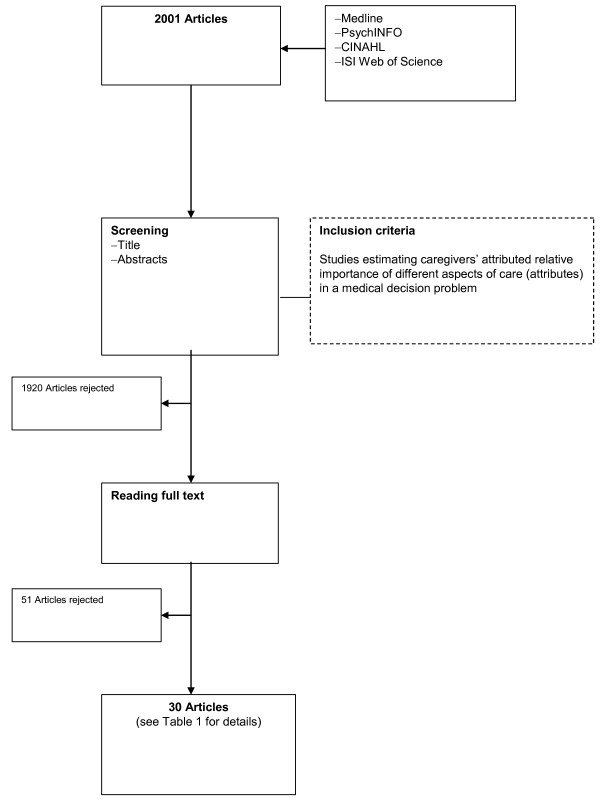
Study flow.

The searches retrieved 2001 records. Full papers of 81 potentially relevant studies were obtained. In total 51 articles did not meet the inclusion criteria and were excluded after reading the full reports, leaving 30 reports published between 1983 and 2005 for evaluation. (See flowchart in the Figure 1) The salient features of included studies are shown in the Table 1.

### General aspects

Although the first study was published in 1983, 24 studies (84%) were published after 1995.

Twenty-seven out of thirty studies examined decision behaviour of medical experts [15,17-42]. In half of the studies more than one type of respondent was surveyed.

Twenty-eight different medical problems were addressed. Twenty-two (73%) studies examined treatment decisions. Eleven studies (37%) asked for a preferred diagnostic decision, sometimes (6 studies) in combination with a treatment decision.

### Objectives

Ten studies (33 percent) aimed at describing decision preferences of specific groups of participants [20,24,26,27,29,30,32,38,43,44] and nine studies (30 percent) described decision preference differences between groups [18,25,28,31,34,36,37,40,41]. Three studies explored the consistency of decisions between groups of experts [15,23,45] and two studies examined change of preferences after an intervention [22,35]. Only six studies (20 percent) compared decision behaviour against some sort of empirical reference such as a guideline [21,39,42] (n = 3), actual patient data [17,19] (n = 2) or the result of a clinical study [33].

### Design

The median number of attributes was 6.5 (inter quartile range IQR 4–9, range 2–15). In 20 studies (67%) the selection of attributes was based on information like the literature [17,20,27,28,31,32,34,35,37,40,43,45] (12 studies), expert opinion (7 studies) or guidelines (1 study). In five studies patient files [15,21,24,33,41] were used to construct the vignettes.

The median number of vignettes was 25 (IQR 16–32), ranging from 3 to 130.

Authors used several response modes for the vignettes. In eight cases they used more than one response mode. In 23 cases authors used a rating procedure [15,18,20-25,27-31,34-37,40-45], where respondents had to rate the relative importance of a given vignette or assign a probability (n = 6) to a diagnosis or outcome [31-33,35-37]. One study used a ranking design, where respondents had to arrange each of the attributes in descending order of importance [24]. In six studies respondents could reply with a yes/no choice [27,30,31,35,38,39]. One study used a conventional discrete choice mode, where respondents, given two or more vignettes, had to select one with the highest likelihood of postoperative recovery [26].

### Analysis

Twenty (67%) studies did not correct for correlated data. Consequently, only ten studies applied some statistical procedure to account for this correlation within the data [15,22,26,32-37,41].

## Discussion

This review has two main findings. First, studies of medical choice and judgement are regularly used in the medical field to explore healthcare providers' decision behaviour or preferences. Second, we found a broad spectrum of different methods, and both design and analysis were suboptimal in some cases.

### Cognitive burden/complexity

One fourth of our studies either contained vignettes with more than nine attributes or compiled sets of over forty vignettes in the same experiment. Empirical evidence showing that these figures are too high is scarce and there is much controversy particularly about the number of vignettes [46]. From a cognitive psychological point of view both figures appear to be very high and could bias the results. This bias typically occurs because respondents are unable to integrate and process large information quantities provided simultaneously, or because respondents lose attention when sifting through too many vignettes. However, evidence suggests that more attributes, more choice options and more vignettes decrease response reliability, but do not bias mean responses [46]. As a rule of thumb, the number of attributes per vignette should not exceed six to eight [47-49]. There is much opinion and controversy about maximally allowed number of vignettes, but little rigorous evidence [46]. A re-analysis of 21 commercial studies suggests a maximum of 20 vignettes [48] and a review of discrete choice experiments evaluating healthcare shows that the number of vignettes seldom exceeds 16 [49]. Furthermore, the majority of studies either used a ranking or rating response mode. These two modes imply very strong assumptions about human cognitive abilities making it more likely that measures will be biased and invalid [50]. Consequently, we therefore recommend the choice based approach.

### Validity, usefulness of study objectives

In contrast to applications in marketing research where the main topic of a study is to identify opinions regarding a new product, we would be particularly interested to learn about the correctness of care givers' weighting of the value of clinical information in decisions. While there is no normative benchmark for a "correct" product there is usually one in medical judgement if clinical studies are available. For example, if the results of a study on medical choice and judgement showed that physicians consistently attribute high weights to relatively uninformative lab test but instead undervalue the informativeness of cues from clinical examination they would hint at something that needed to be improved perhaps with an educational intervention. Also the method would allow assessing the change in preferences after intervening with educational measures.

Most studies did not compare the attributed weights to some sort of normative benchmark such as the results of a clinical study. We only found one out of 30 studies that actually examined this and another five that used a further normative reference (guidelines or patient files). In absence of a normative benchmark these studies leave it to the reader to approve or disapprove the results. Moreover, assessment of discrepancies between different groups of participants has the problem that these could be explained by different clinical circumstances or other factors rather than group specific differences. On the other hand there are medical situations in which views about optimal choices are controversial. In these situations studies that do not compare caregivers' decision behaviour (or preferences) to some norm may still be useful in that they allow the examination of present opinions.

### Statistical model

The majority of studies did not account for correlated data in the analysis. Correlated data occur because each respondent assesses different vignettes. Not accounting for this leads to too small estimates of the standard deviations for an attribute and can mimic a statistically significant association where in fact there is none. Unfortunately, guidelines on the conduct of conjoint analyses have not yet reached consensus about the optimal way to analyse correlated data.

### Limitations

What are the limitations of this review? We think that the search and appraisal procedures were reliable. However, sometimes classifications were difficult to make because of unclear descriptions in the article. We did not contact authors to clarify these uncertainties. Second, there have been two prominent methods of constructing linear models of medical judgements, each with their own literature and set of advocates. These are conjoint analysis, developed in the 1970s to study preference and choice[1], and judgement analysis, also called social judgement theory, developed in the 1950s from Brunswik's lens model[2,3]. In this review we did not make a distinction between the two methods because there is substantial overlap in methodology. Arguably this is a weakness of our study. However, since we were interested in providing an overview of all studies that examined medical decisions of care givers using cue weightings from answers to structured vignettes applying all sorts of different methods, we feel that our approach has its own merit.

### Future research

Our review indicates that current applications of conjoint and judgment analysis in the medical field remain suboptimal in some instances. We think that researchers should consider our propositions to ensure internal validity. Moreover we believe that studies investigating care givers' judgements are most valuable if they allow comparisons with some norm and if they include an assessment of deviations from that norm. Our review only found few such investigations. From a more methodological point of view we agree with a statement in a recent editorial that research is required to learn whether individuals do behave in reality as they state in a hypothetical context. [51]

## Conclusion

We believe that studies of medical choice and judgement offer many attractive and new insights into medical action. Provided that both methods and application evolve they offer a unique opportunity to improve quality of care.

## Competing interests

The authors declare that they have no competing interests.

## Authors' contributions

AGHK conceived of the study and LMB obtained funding. AGHK and LMB designed the study, supervised the work and drafted the manuscript. AM and AB carried out the data extraction. AM, AB, UH and GtR participated in the design of the study and gave important conceptual input. All authors read and approved the final manuscript.

## Pre-publication history

The pre-publication history for this paper can be accessed here:


